# Urogenital Abnormalities in Adenosine Deaminase Deficiency

**DOI:** 10.1007/s10875-020-00777-8

**Published:** 2020-04-19

**Authors:** Roberta Pajno, Lucia Pacillo, Salvatore Recupero, Maria P. Cicalese, Francesca Ferrua, Federica Barzaghi, Silvia Ricci, Antonio Marzollo, Silvia Pecorelli, Chiara Azzari, Andrea Finocchi, Caterina Cancrini, Gigliola Di Matteo, Gianni Russo, Massimo Alfano, Arianna Lesma, Andrea Salonia, Stuart Adams, Claire Booth, Alessandro Aiuti

**Affiliations:** 1 Department of Pediatrics, Endocrine Unit, IRCCS San Raffaele Scientific Institute, Via Olgettina, 60, 20132 Milan, Italy; 2 Department of Pediatrics, “Pietro Barilla” Children Hospital, University of Parma, via Gramsci, 14 Parma, Italy; 3 Unit of Immune and Infectious Diseases, Scientific Institute for Research and Healthcare (IRCCS) Childrens’ Hospital Bambino Gesù, University Department of Pediatrics (DPUO), Rome, Italy; 4 Department of Systems Medicine, University of Rome Tor Vergata, Rome, Italy; 5 Pediatric Immunohematology and Stem Cell Program, IRCCS San Raffaele Scientific Institute, Via Olgettina, 60, 20132 Milan, Italy; 6 San Raffaele Telethon Institute for Gene Therapy (SR-Tiget), IRCCS San Raffaele Scientific Institute, Milan, Italy; 7 Division of Pediatric Immunology, Department of Health Sciences, University of Florence and Meyer Children’s Hospital, Florence, Italy; 8 Pediatric Hematology-Oncology Unit, Department of Women’s and Children’s Health, University of Padova, Via Giustiniani 3, 35128 Padova, Italy; 9 Department of Pediatric Surgery, Ospedale dei Bambini – Spedali Civili, Brescia, Piazzale Spedali Civili 1, 25123 Brescia, Italy; 10 Division of Experimental Oncology/Unit of Urology, URI, IRCCS Ospedale San Raffaele, Milan, Italy; 11 Unit of Pediatric Surgery, Department of Urology, IRCCS Ospedale San Raffaele, Milan, Italy; 12 Università Vita-Salute San Raffaele, Milan, Italy; 13 SIHMDS-Haematology, Great Ormond Street Hospital for Children, London, UK; 14 Department of Paediatric Immunology, Great Ormond Street Hospital, London, UK

**Keywords:** ADA-SCID, puberty, pubertal development, cryptorchidism, undescended testis, urogenital abnormalities

## Abstract

**Background:**

Improved survival in ADA-SCID patients is revealing new aspects of the systemic disorder. Although increasing numbers of reports describe the systemic manifestations of adenosine deaminase deficiency, currently there are no studies in the literature evaluating genital development and pubertal progress in these patients.

**Methods:**

We collected retrospective data on urogenital system and pubertal development of 86 ADA-SCID patients followed in the period 2000–2017 at the Great Ormond Street Hospital (UK) and 5 centers in Italy. In particular, we recorded clinical history and visits, and routine blood tests and ultrasound scans were performed as part of patients’ follow-up.

**Results and Discussion:**

We found a higher frequency of congenital and acquired undescended testes compared with healthy children (congenital, 22% in our sample, 0.5–4% described in healthy children; acquired, 16% in our sample, 1–3% in healthy children), mostly requiring orchidopexy. No urogenital abnormalities were noted in females. Spontaneous pubertal development occurred in the majority of female and male patients with a few cases of precocious or delayed puberty; no patient presented high FSH values. Neither ADA-SCID nor treatment performed (PEG-ADA, BMT, or GT) affected pubertal development or gonadic function.

**Conclusion:**

In summary, this report describes a high prevalence of cryptorchidism in a cohort of male ADA-SCID patients which could represent an additional systemic manifestation of ADA-SCID. Considering the impact urogenital and pubertal abnormalities can have on patients’ quality of life, we feel it is essential to include urogenital evaluation in ADA-SCID patients to detect any abnormalities, initiate early treatment, and prevent long-term complications.

**Electronic supplementary material:**

The online version of this article (10.1007/s10875-020-00777-8) contains supplementary material, which is available to authorized users.

## Introduction

Severe combined immunodeficiency due to adenosine deaminase deficiency is a rare autosomal recessive disease (ADA-SCID, OMIM # 102,700) caused by mutations in the gene encoding the enzyme ADA type 1, resulting in impairment of the purine salvage pathway [1–3]. This defect in purine metabolism primarily affects lymphocyte development and function resulting in varying degrees of immune deficiency [4].

Several studies demonstrate that ADA-SCID is a systemic disease, and thanks to improved survival, an increasing number of non-immune manifestations are being recognized and reported [1–5].

At present, no study describes abnormalities in the development of genitalia or in the pubertal progression of ADA-SCID patients treated for their underlying immune disorder.

## Methods

In this report, we describe data collected retrospectively on the urogenital system and pubertal development of 86 ADA-SCID patients followed in the period 2000–2017: 51 males and 35 females with an age range from 4 months to 30 years were included in this analysis (Table 1). Patients were from different ethnicities, and there was a high prevalence of consanguinity (51%). Previous treatments included enzyme replacement therapy (PEG-ADA ERT), gene therapy (GT), or allogeneic bone marrow transplantation (BMT) as single therapy or given in various combinations (Table 1).

**Table 1:** Sample description, sex, origin, parents’ consanguinity, ADA-SCID treatment, and years of follow-up

N°	sex	Origin	C	ADA mutation	Treatment	Years of follow-up § (age)
1	F	South America/Hispanic	Yes	Compound heterozygous, c.320 T > C, p.L107P/c.632G > A, p.R211H	Haploidentical BMT° ➔ GT^1^➔PEG-ADA	15 (3–18 y)
2	M	South America/Hispanic	No	Compound heterozygous, c.221G > T, p.G74V/c.845G > A, p.R282Q	Haploidentical BMT° ➔ GT^1^	14 (1–15 y)
3	F	Arabic/White	Yes	Homozygous c.845G > A, p.R282Q	Haploidentical BMT° ➔ PEG-ADA ➔ GT^1^	13 (1–14 y)
4	F	Arabic/White	Yes	Compound heterozygous, c.646G > A, p.G216R/c.956_960delAAGAG; p.E319GfsX3	PEG-ADA ➔ GT^1^	11 (1–12 y)
5	M	Europe/White	Yes	Homozygous c.632G > A, p.R211H	PEG-ADA ➔ GT^1^	12 (5–17 y)
6	M	Europe/White	No	Compound heterozygous, c.646G > A, p.G216R/c.872C > T, p.S291L	PEG-ADA ➔ GT^1^	11 (0–11 y)
7	M	Europe/White	No	Homozygous c.478 + 2 T > **C**	PEG-ADA ➔ GT^1^	10 (1–11 y)
8	F	Arabic/White	Yes	Homozygous c.646G > A, p.G216R	Haploidentical BMT° ➔ PEG-ADA ➔ GT^1^	8 (0–8 y)
9	M	South America/Hispanic	Yes	Homozygous c.632G > A, p.R211H	PEG-ADA ➔ GT^1^	9 (0–9 y)
10	M	North America/White	No	Compound heterozygous, c.646G > A, p.G216R/c.956_960delAAGAG; p.E319GfsX3	PEG-ADA ➔ GT^1^	9 (1–10 y)
11	M	South Asia	Yes	Homozygous c.606 + 5G >? (Exon6, splice donor site + 5— no more data available)	PEG-ADA ➔ GT^1^	9 (0–9 y)
12	M	North America/White	No	Compound heterozygous, c.646G > A, p.G216R/Exon10, deletion + 6 c.975 + 6Tdel	PEG-ADA ➔ GT^1^	8 (6–14 y)
13	F	Africa/White	No	Homozygous: c.466C > T, p.R156C	PEG-ADA ➔ GT^1^	8 (2–10 y)
14	M	Africa/Black	No	Homozygous, c.7C > T, p.Q3X	PEG-ADA ➔ GT^1^	6 (2–8 y)
15	M	Africa/Black	Yes	Homozygous, c.881C > A, p.T294K	PEG-ADA ➔ GT^1^ ➔ MSD BMT^2^	4 (1–5 y)
16	M	Arabic/White	Yes	Homozygous, c.956_960delAAGAG, p.E319GfsX3	PEG-ADA ➔ GT^1^	5 (2–7 y)
17	F	European/White	No	Compound heterozygous, c.632G > A, p.R211H/c.646G > A, p.G216R	PEG-ADA ➔ GT^1^	2 (0–2 y)
18	M	Europe/Hispanic	No	Compound heterozygous, c.467G > A, p.R156H / c.646G > A, p.G216R	PEG-ADA ➔ GT^1^ ➔ MUD BMT*	3 (2–5 y)
19	M	Europe/White	Yes	Compound heterozygous, c.385G > A, p.V129M /(second mutation not identified)	PEG-ADA	16 (14–30 y)
20	F	Europe/White	Unk	Homozygous, c.385G > A, p.V129M	PEG-ADA	23 (4–27 y)
21	F	Europe/White	No	Homozygous, c.499delG**,** pV167P	PEG-ADA	12 (6–18 y)
22	M	Europe/White	Yes	Homozygous, c.632G > A, p.R211H	PEG-ADA	17 (3–20 y)
23	M	Europe/White	Yes	Homozygous, c.632G > A, p.R211H	PEG-ADA ➔ MSD BMT^3^	10 (0–10 y)
24	M	Europe/White	Unk	Homozygous, c.632G > A, p.R211H	PEG-ADA ➔ MSD BMT°	6 (5–10 y)
25	M	South America/Hispanic	No	Homozygous, c.845G > A, p.R282Q	PEG-ADA ➔ MUD BMT°	1 (0–1 y)
26	F	Europe/White	Unk	Exon 3, insertion (no more data available)	PEG-ADA ➔ MSD BMT°	14 (0–14 y)
27	M	Europe/White	No	Compound heterozygous, c.466C > T, p.R156C/c.955_959GAAGA, p.E320GfsX3	PEG-ADA ➔ MUD BMT°	13 (1–14 y)
28	M	Europe/White	Unk	ND	Haploidentical BMT*	15 (0–15 y)
29	F	Europe/White	Unk	ND	PEG-ADA ➔ MUD BMT*	11 (0–11 y)
30	F	South Asia	Unk	Homozygous, c.424C > T, p.R142X	PEG-ADA ➔ MSD BMT°	13 (0–13 y)
31	M	Unk	Unk	Homozygous, c.424C > T, p.R142X	PEG-ADA ➔ MSD BMT°	17 (0–17 y)
32	F	Africa/Black	Yes	Homozygous, c.7C > T, p.Q3X	PEG-ADA ➔ MSD BMT°	18 (0–18 y)
33	M	Europe/White Irish	Unk	Homozygous, c.646G > A, p.G216R	PEG-ADA ➔ MFD BMT°	17 (0–17 y)
34	F	South Asia	Yes	ND	PEG-ADA ➔ MFD BMT°	18 (0–18 y)
35	F	Europe/White	Unk	Compound heterozygous, c.363-1G > C/c.364G > A, p.G122R	PEG-ADA ➔ MUD BMT^4^	18 (0–18 y)
36	M	Europe/White	Yes	Homozygous, c.646G > A, p.G216R	PEG-ADA ➔ MFD BMT°	17 (0–17 y)
37	F	Europe/White	Yes	ND	PEG-ADA ➔ MSD BMT°	16 (0–16 y)
38	F	Europe/White Irish	Yes	Homozygous, c.646G > A, p.G216R	PEG-ADA ➔ MFD BMT°	15 (0–15 y)
39	M	Africa/Black	Yes	Homozygous, c.7C > T, p.Q3X	PEG-ADA ➔ GT^5^	17 (0–17 y)
40	M	Africa/Black	No	Homozygous, c.7C > T, p.Q3X	PEG-ADA ➔ MFD BMT°	14 (0–14 y)
41	M	Africa/Black	Yes	Homozygous, c.646G > A, p.G216R	PEG-ADA ➔ MSD BMT°	13 (0–13 y)
42	F	Africa/Black	No	Homozygous, c.7C > T, p.Q3X	PEG-ADA ➔ MUD BMT^6^	14 (0–14 y)
43	M	South Asian heritage	Yes	Homozygous, c.716G > A, p.G239D	PEG-ADA ➔ MSD BMT°	12 (0–12 y)
44	M	Europe/White	No	Compound heterozygous, c.367delG, p.D123TfsX10/c.956_960delAAGAG; p.E319GfsX3	PEG-ADA ➔ GT^5^	13 (0–13 y)
45	F	Europe/White	No	Compound heterozygous, c.467G > A, p.R156H/c.478 + 1G > A	PEG-ADA ➔ GT (first)^5^ ➔ GT (second)^7^	13 (2–15 y)
46	M	South Asia	Yes	Homozygous, c.716G > A, p.G239D	PEG-ADA ➔ MSD BMT°	11 (0–11 y)
47	F	Europe/White	Yes	Homozygous, c.646G > A, p.G216R	PEG-ADA ➔ MFD BMT°	11 (1–12 y)
48	M	Arabic /white	Yes	Homozygous, c.956_960delAAGAG; p.E319GfsX3	PEG-ADA ➔ GT^5^	8 (0–8 y)
49	M	Arabic/White	Yes	Homozygous, c.385G > A, p.V129M	PEG-ADA ➔ MSD BMT°	5 (1–6 y)
50	M	Europe/White	No	ND	PEG-ADA ➔ GT^5^	3 (1–4 y)
51	F	Africa/Black	Yes	Homozygous, c.7C > T, p.Q3X	PEG-ADA ➔ MSD Cord°	10 (0–10 y)
52	M	Africa/Black	Yes	Homozygous, c.646G > A, p.G216R	PEG-ADA ➔ GT^5^	11 (0–11 y)
53	F	South Asia	Yes	Homozygous, c.646G > A, p.G216R	PEG-ADA ➔ MMUD Cord^8^	9 (0–9 y)
54	F	South Asia	Yes	Homozygous, c.703C > T, p.R235W	PEG-ADA ➔ MMUD Cord^8^	10 (0–10 y)
55	M	Arabic/White	Yes	Homozygous, c.428dupA, p.D143EfsX28	PEG-ADA ➔ MUD Cord^8^	3 (0–3 y)
56	M	Europe/White	No	Compound heterozygous, c.466C > T, p.R156C/c.646G > A, p.G216R	PEG-ADA ➔ GT^5^ ➔ HSCT^7^	12 (1–13 y)
57	M	South Asia	Yes	Homozygous, c.646G > A, G216R	PEG-ADA ➔ MUD Cord ➔ MUD PBSC^9^	9 (0–9 y)
58	F	South Asia	Yes	Homozygous, c.716G > A, p.G239D	PEG-ADA ➔ MFD BMT°	7 (0–7 y)
59	M	Europe/White	No	Compound heterozygous, c.955-958delGAAG, p.E320RfsX6/c.1078 + 2 T > A	PEG-ADA ➔ GT (first) ➔ GT (second)	8 (4–12 y)
60	F	Arabic/White	Yes	Homozygous, 1079-15 T > A	PEG-ADA ➔ MUD PBSC^5^	3 (1–4 y)
61	M	Arabic/White	Yes	Homozygous, c.385G > A, p.V129M	PEG-ADA ➔ MFD BMT^10^	4 (0–4 y)
62	M	Europe/White Irish	Yes	Homozygous, c.646G > A, p.G216R	PEG-ADA ➔ MFD BMT^0^	7 (0–7 y)
63	M	Africa/Black	Yes	Homozygous, c.7C > T, p.Q3X	PEG-ADA ➔ GT^7^	3 (4–7 y)
64	M	South Asia	Yes	Homozygous, c.646G > A, p.G216R	PEG-ADA ➔ GT^7^	7 (0–7 y)
65	F	Europe/White	No	Compound heterozygous, c.646G > A, p.G216R/c.955_959GAAGA, p.E320GfsX3	PEG-ADA ➔ GT^7^	5 (0–5 y)
66	F	South Asia	Yes	Homozygous, c.646G > A, p.G216R	PEG-ADA ➔ GT^7^	5 (0–5 y)
67	M	Arabic/white	No	Compound heterozygous, c.976-1G > C/c.302G > T, p.R101L	PEG-ADA ➔ GT (first) ^7^ ➔ GT (second) ^7^	9 (1–10 y)
68	M	Africa/Black	No	Homozygous, c.7C > T, p.Q3X	PEG-ADA ➔ GT^7^	4 (0–4 y)
69	F	Europe/White	No	Compound heterozygous, c.872C > T, p.S291L/c.986C > T, p.A329V	PEG-ADA ➔ GT^7^	4 (1–5 =y)
70	M	Africa/Black	No	Homozygous: c.7C > T, p.Q3X	PEG-ADA ➔ GT^7^	4 (0–4 y)
71	M	Africa/Black	No	Compound heterozygous, c.603C > G, p.Y201X/c.632G > A, p.R211H	PEG-ADA ➔ GT^7^	4 (0–4 y)
72	F	Africa/Black	No	Homozygous, c.7C > T, p.Q3X	PEG-ADA ➔ GT^7^	4 (9–13 y)
73	F	Europe/White Irish	Yes	Homozygous, c.646G > A, p.G216R	PEG-ADA ➔ GT^7^	2 (0–2 y)
74	M	Europe/White	No	Homozygous, c.646G > A, p.G216R	PEG-ADA ➔ MFD BMT ➔ MSD BMT°	2 (0–2 y)
75	M	Unk	No	Compound heterozygous, c.320 T > C, p.L107P/c.632G > A, p.R211H	PEG-ADA ➔ GT^7^	1 (1–2 y)
76	F	Africa/Black	Yes	Homozygous, c.7C > T, p.Q3X	PEG-ADA ➔ GT^7^	3 (0–3 y)
77	M	Europe/White Irish	No	Homozygous, c.646G > A, p.G216R	PEG-ADA ➔ GT^7^	2 (0–2 y)
78	M	Europe/White	Yes	Compound heterozygous, c.310C > A, p.P104T/c.646G > A, p.G216R	PEG-ADA ➔ GT^7^	2 (0–2 y)
79	F	Europe/White	Yes	Compound heterozygous, c.43C > G, p.H15D/c.757_758dupCG	PEG-ADA ➔ GT^7^	2 (0–2 y)
80	F	Europe/White	No	Homozygous, c.646G > A, p.G216R	PEG-ADA ➔ GT^7^	2 (0–2 y)
81	M	Africa/White	No	Homozygous, c.704G > A, p.R235Q	PEG-ADA ➔ GT^7^	1 (2–3 y)
82	M	Europe/White Irish	Yes	Homozygous, c.646G > A, G216R	PEG-ADA ➔ GT^7^	2 (0–2 y)
83	F	Europe/White	No	Homozygous, c.320 T > C, p.L107P	PEG-ADA ➔ GT^7^	0
84	M	Africa/Black	Yes	Homozygous, c.7C > T, p.Q3X	PEG-ADA ➔ GT^7^	1 (0–1 y)
85	F	Europe/White Irish	Yes	Homozygous, c.646G > A, G216R	PEG-ADA ➔ GT^7^	0
86	F	Europe/White-Africa/Black	No	Compound heterozygous, c.482G > A, p.W161X/c.1078 + 2 T > A	PEG-ADA ➔ GT^7^	0

*C* parents’ consanguinity, *Unk* unknown, *§* years of follow-up are considered time from the first diagnostic test available to the last. In parentheses, age of the diagnostic test available–age of the last diagnostic test available. *ND* not done, *BMT* bone marrow transplantation, *GT* gene therapy, *MSD* BMT from matched sibling donor, *MFD* BMT from matched family donor, *MUD* BMT from matched unrelated donor, *MMUD* BMT from mismatched unrelated donor, *PBSC* peripheral blood stem cells, *Cord* cord blood cells

In the column treatment superscript numbers:

*Unknown

^0^No conditioning agents

^1^Busulfan (single agent, non myeloablative)

^2^Reduced toxicity regimen Treo/Flu

^3^Reduced intensity conditioning (RIC) Bu/Flu

^4^RIC Flu/Melph/ATG

^5^Melphalan (single agent)

^6^RIC Flu/Melph/Campath

^7^Low-dose busulfan (AUC ~ 20)

^8^Myeloablative conditioning (MAC) Treo/Cy

^9^MAC Treo/Flu

^10^Campath (single agent)

Patients in our cohort received immunological follow-up in five hospitals: 23 patients have been followed at our center, 1 patient at Bambin Gesù Hospital in Rome, 2 patients at Hospital Meyer in Florence, 1 patient at Hospital in Padova, and 59 patients in Great Ormond Street Hospital, London. Italian hospitals are part of the AIEOP (Associazione Italiana di Ematologia e Oncologia Pediatrica) and IPINET (Network Italiano Immunodeficienze Primitive) network.

Patients or their guardians provided written informed consent according to local consent procedures. This report was performed in accordance with the ethical standards of the institutional research committees and with the 1964 Helsinki declaration and its later amendments.

We collected the information registered during the immunological follow-up. Medical history, clinical data, routine blood tests, and ultrasound scans performed as part of patients’ follow-up were recorded in patients’ notes. If patients presented with clinical issues during the follow-up, additional investigations were performed. In male patients, we documented the number of patients with cryptorchidism, whether cryptorchidism was unilateral or bilateral, congenital (testis not present in the scrotum from birth by 3 months of age), or acquired (testis that was originally present in the scrotum at birth but ascends later) [6] or if the cryptorchidism solved spontaneously or required orchiopexy, the age of surgery, and any recurrences. We registered any urological malformation associated with cryptorchidism and the presence of phimosis and requirement for circumcision. Analyzing the complete cohort of patients, pubertal progression was evaluated at every clinical evaluation available for follow-up in both males and females. We documented the age of spontaneous puberty and every case of precocious or late puberty. Female patients underwent abdominal US scan as part of the follow-up; we documented data of any alteration of gonads at US scan. As markers of puberty, the following blood tests were performed in the majority of patients: luteinizing hormone (LH), follicle-stimulating hormone (FSH), testosterone (male patients), or estradiol (female patients). Analysis of these biomarkers (measured using fluorimetric methods) together with clinical evaluation of puberty allows evaluation of the hypothalamus-pituitary-gonad axis function. Moreover, if these hormones are evaluated in the first 3–6 months of life, it is possible to identify mini-puberty during which LH and FSH increase as it happens during puberty. This is a physiologic hormonal fluctuation without clinical manifestations associated with sex steroids rising to level reached in early-middle pubertal levels, without peripheral effects. If mini-puberty is identified with blood tests, it suggests normal hypothalamus-pituitary-gonad axis function. It has been hypothesized that this hormonal phase has a role in physiologic descent of testis in the first year of life in transient congenital cryptorchidism [6–9].

## Results

Regarding genital development, results differed between males and females.

Of 51 male patients, 11 (22%) presented congenital undescended testes; of those, 6 (54.5%) were bilateral and 7 (63.6%) required orchidopexy, respectively (Table 2). Eight out of 51 (16%) presented acquired undescended testes and among these 3/8 were bilateral and 7/8 required orchidopexy. None of the patients presenting with undescended testes were born at < 36 weeks gestation. Six of 11 patients with congenital undescended testes had consanguineous parents (54%, Tables 1 and 2). Among other urogenital abnormalities seen, 3/51 patients presented with inguinal hernia requiring surgical intervention, 6/51 presented micropenis of whom 4 had associated cryptorchidism, and one subject had posterior urethral valves. Nine out of 51 (18%) presented phimosis, and 5/9 were treated with circumcision (Table 2).

**Table 2:** Male sample, urogenital abnormalities, pubertal development, hormonal tests, and testis US scan

N°	WG	CUT	AUT	Treatment of undescended testes	Other urogenital diseases	Puberty	Testis structure at US scan	Hypothalamus-pituitary-gonads axis
2	≥ 37	Left	Right (9 y)	Left orchidopexy 2 y and 7 months Right orchidopexy 9 y and 11 months. No relapse	Phimosis	Pubescent at 15 y (G2P4)	Dyshomogeneous (hyporeflectant areas) since 14 y	Physiologic activation
5	36	Right	No	Right orchidopexy. No relapse	1) Phimosis 2) inguinal hernia	Pubescent at 15 y G5	Normal	Physiologic activation
6	36 + 1	Bilat	No	Bilateral orchidopexy 2 y and 3 months. No relapse	Phimosis	Prepubescent at 10 y	Normal	Not activated
7	≥ 37	No	No	NA	Phimosis	Prepubescent at 11 y	ND	Not activated
9	≥ 37	No	Right (6 y)	Right orchidopexy 7 y and 2 months. No relapse	Phimosis	Prepubescent at 9 y	Hyporeflectant areas since 13 y	Not activated
10	≥ 37	no	No	NA	No	Prepubescent at 8 y	ND	Not activated
11	≥ 37	Bilat	No	Gonadoreline not effective. Bilateral orchidopexy 3 y. Bilateral relapse Gonadoreline not effective. Left orchidopexy 5y 6mo. Bilateral relapse 7 y	1) Phimosis 2) Micropenis 3) Posterior urethral valve left megaureter	Normal mini-puberty Prepubescent at 9 y	ND	Not activated
12	32	no	No	NA	No	Prepubescent at 13 y	Homogenous but less reflectant since 11 y	Not activated
14	≥ 37	Left	Bilat (7 y)	Congenital undescended testes spontaneously solved Bilateral orchidopexy 8 y. No relapse	Phimosis	Prepubescent at 8 y	Normal	Not activated
15	≥ 37	No	No	No	Phimosis	Prepubescent at 5 y	Homogenous but less reflectant since 11 y	Not activated
16	≥ 37	No	Bilat (3 y)	Bilateral orchidopexy 4y. Left relapse 5 y	No	Prepubescent at 7 y	Normal	Not activated
19	≥ 37	No	Right	Gonadoreline, effective. Right relapse right orchidopexy 13y + hernioplastic. No relapse	Inguinal hernia	Pubescent at 30 y G5	Normal	Not activated
22	≥ 37	No	Left (2 y)	Spontaneously solved. No relapse	Inguinal hernia	Pubescent at 15 y G4	ND	Not activated
24	≥ 37	No	Bilat (5 y)	Bilateral orchidopexy 5 y. No relapse	No	Pubescent—early onset **(**at 11 y G4)	Hyperreflectant spots (seminiferous tubule fibrosis)	Physiologic activation
28	Unk	No	No	NA	Micropenis	CDGP; 15 y after testosterone: G2P3A1	Normal	ND
31	> 37	Bilat	No	Unk	Micropenis	CDGP; at 17 y: G3P3A2	Normal	Physiologic activation
33	Unk	No	No	NA	Micropenis	Unk	ND	ND
36	> 37	No	No	NA	No	CDGP; at 17 y after testosterone: G4P4A2	ND	ND
40	> 37	No	No	NA	No	Prepubescent at 14 y	Normal	ND
43	> 37	Bilat	No	2 y bilateral orchidopexy	No	Pubescent at 12 y (G4P3A2), early onset	ND	ND
57	> 37	Bilat	No	Not done yet—performing follow-up	Micropenis	Normal mini-puberty Prepubescent at 9 y	ND	ND
59	>37	Right	No	11 y right orchidopexy	Phimosis	Prepubescent at 11 y	ND	ND
62	Unk	Left	No	18 months left orchidopexy	No	Prepubescent at 7 y	Normal	ND
68	> 37	No	Right	4 y right orchidopexy	No	Prepubescent 4 y and 7 months	Normal	Not activated
82	> 37	Bilat	No	Not done yet—performing follow-up	Micropenis Undervirilized scrotum	Suspect hypogonadism hypogonadotropic (no mini-puberty) Prepubescent 1 y and 7 months	ND	ND

Only patients with urogenital abnormalities or alteration in puberty or patients who performed hormonal test/US testis scan are included in the table

Pubertal stage was evaluated with Tanner stage. Hypothalamus-pituitary-gonads axis evaluation: physiologic activation means we registered LH values > 1 mUI/ml, FSH values > 2 mUI/ml and < 10 mUI/ml, testosterone or estradiol levels adequate for age; not activated means LH values < 1 mUI/ml, FSH values < 2 mUI/ml, testosterone not detectable; normal mini-puberty means LH and FSH values similar to puberty values

*WG* week gestation, *UT* undescended testis, *NA* not applicable, *ND* not done, *UNK* unknown. Cryptorchidism: *CUT* congenital undescended testis, *AUT* acquired undescended testis, in brackets the age of diagnosis, *Bilat* bilateral, *CDGP* constitutional delay of growth and puberty

Abdominal US scans performed in 10/35 female patients were normal with no abnormalities documented in ovaries, uterus, or vagina (Table 3).

**Table 3:** Female sample, urogenital abnormalities at pelvic US scan, pubertal development, and hormonal tests

N°	Pelvic us scan	Other urogenital disease	Pubertal stage	Precocious puberty	Treatment with GnRH agonist	Delayed puberty	Hypothalamus-pituitary-gonads axis
3	Normal	No	Pubescent 15 y TS V RM	No	No	No	ND
4	Normal	No	Pubescent 12 y TS V RM	Yes (8 y)	Yes (8–11 y)	No	ND
13	Normal	No	Pubescent 10 y, B4 P2–3	No	No	No	Physiologic activation
17	Normal	No	Prepubescent 3 y	No	No	No	ND
20	Normal	No	Pubescent RM	No	No	No	Physiologic activation
21	Normal	No	Pubescent IM#	Yes (8 y)	No	No	Physiologic activation
42	Normal	No	Pubescent 14 y, RM	No	No	No	Physiologic activation
45	Normal	Polycystic kidney disease	Pubescent at 15 y, RM	No	No	No	ND
53	Normal	No	Pubescent at 9 y, A1P2B3	Yes (9 y)	Yes (9 y–ongoing)	No	Normal

*ND* not done, *TS* Tanner stage, *RM* regular menses, *IM* irregular menses

Only patients who performed US pelvic scan and/or hormonal tests were included in this table (for complete female sample see table in electronic supplemental material). In the column precocious puberty, the age of onset is reported in brackets

# patient n° 21 presented irregular menstrual cycles with prolonged periods of amenorrhea associated with hyperinsulinism, hirsutism, and hyperandrogenism. Polycystic ovary syndrome was suspected, and the patient was treated with cyproterone acetate and transdermal estradiol

In terms of pubertal development, data were available for 33 females and 48 males. In the overall population 28/81 had achieved puberty and 52/81 are still prepubescent (aged less than 14 years). Among female patients, 51.5% are still prepubescent (age ≤ 10 years) while 47.0% presented spontaneous pubertal progression (Table 3). Among these, 3/16 presented early onset of puberty (at 8 years) and 2/3 were treated with gonadotropin-releasing hormone agonists. Among the male patients (Table 2), 73% are still prepubescent (age < 14 years). Nine patients presented spontaneous pubertal development of whom 2 showed early onset of puberty (at 9 years). Three patients presented delayed onset of puberty but appropriate progression (constitutional delay in growth and puberty) of whom 2 were treated with testosterone inducing the onset of puberty.

Hormonal data are available in 20 patients (Table 4). In 9 pubescent patients (5 females, 4 males) hormonal tests showed physiologic activation of the hypothalamic-pituitary-gonadal axis. In 11 prepubescent patients (11 males), LH, FSH, and testosterone or estradiol resulted low. None of the patients had raised FSH values. No patient with delayed puberty presented hypogonadotropic hypogonadism (HH) although one patient with delayed puberty was not investigated (patient n° 40—age 14 years). Three patients with micropenis and bilateral cryptorchidism underwent blood tests within the first 6 months of life (during mini-puberty), and 2 presented physiologic activation of hypothalamic-pituitary-gonadal axis (Table 2). In one patient, HH was suspected, and testosterone treatment was commenced (the patient is 1 year old).

**Table 4:** Puberty and hormonal tests in male and female patients

N°	Sex	Pubertal stage	Hypothalamus-pituitary-gonads axis
2	M	Pubescent at 15 y (G2P4)	Physiologic activation
5	M	Pubescent at 15 y (G5)	Physiologic activation
6	M	Prepubescent at 10 y	Not activated
7	M	Prepubescent at 11 y	Not activated
9	M	Prepubescent at 9 y	Not activated
10	M	Prepubescent at 8 y	Not activated
11	M	Prepubescent at 9 y	Normal mini-puberty Not activated
12	M	Prepubescent at 13 y	Not activated
13	F	Pubescent 10 y, B4 P2–3	Physiologic activation
14	M	Prepubescent at 8 y	Not activated
15	M	Prepubescent at 5 y	Not activated
16	M	Prepubescent at 7 y	Not activated
19	M	Pubescent at 30 y (G5)	Physiologic activation
20	F	Pubescent, regular menses	Physiologic activation
21	F	Pubescent, irregular menses (polycystic ovary syndrome)	Physiologic activation
24	M	Pubescent at 10 y (G1–2)	Physiologic activation
42	F	Pubescent 14 y, regular menses	Physiologic activation
53	F	Pubescent at 9 y: A1P2B3	Physiologic activation
57	M	Prepubescent at 9 y	Normal mini-puberty Not activated
82	M	Prepubescent 1 y and 7 months	Suspected hypogonadotropic hypogonadism, no mini-puberty (testosterone treatment)

Only patients who performed hormonal tests were included in this table. Pubertal stage evaluated with Tanner stage. Hypothalamus-pituitary-gonads axis evaluation: physiologic activation means we registered LH values > 1 mUI/ml, FSH values > 2 mUI/ml and < 10 mUI/ml, testosterone or estradiol levels adequate for age; not activated means LH values < 1 mUI/ml, FSH values < 2mUI/ml, testosterone or estradiol not detectable; normal mini-puberty means LH and FSH values similar to puberty values

## Discussion

Currently, there are no studies in the literature evaluating genital development or pubertal progression in ADA-SCID patients. No abnormalities of the gonads, uterus, and vagina were detected in the female subgroup, even if these data should be taken with caution since only a minor proportion of female subjects was studied. Therefore, we cannot exclude the association of urogenital abnormalities in female ADA-SCID. Conversely, we identified a high proportion of congenital and acquired undescended testes. In particular, the incidence of congenital undescended testes was higher in our cohort (22%) compared with healthy full-term neonates (0.5–4%, few authors report incidence up to 9%) [6–8]. Moreover, while in the general population 70–80% of undescended testes resolve spontaneously with only 23% requiring orchidopexy, the proportion of ADA-SCID patients eventually requiring orchidopexy was higher, with 64% of finally requiring surgery.

A higher incidence of congenital undescended testes is detected in premature neonates (up to 45%) [6, 7] but all patients with cryptorchidism in our sample were born at term (Table 2). Congenital cryptorchidism is a manifestation of numerous clinical syndromes; the ratio of non-syndromic to syndromic cryptorchidism is described to be greater than 6:1 [7]. In our sample there is high percentage of consanguinity (54% of patients with congenital undescended testes have consanguineous parents, Tables 1 and 2). Given the high rate of consanguinity in our cohort we cannot rule out the possibility of an additional inherited defect accounting for this increased incidence. However, even in patients without consanguineous parents, the incidence remains high compared with the general population (5/51, 10%).

Considering the pathogenesis, cryptorchidism is due to aberrant embryological development. The embryology of testicular descent is complex involving numerous anatomical structures and hormones [6–7]. Androgens are known to play a role in this as HH and panhypopituitarism are associated with bilateral cryptorchidism [9]. Also, the possibility that environmental chemicals interfere with normal reproductive tract development has been raised [7]. We feel we can exclude the hypothesis of HH here as we did not detect a delay in puberty usually associated with HH. Thirty-five percent of our patients entered spontaneous pubertal development and progression with adequate hormone levels; the remaining patients are aged 14 years or less. One can hypothesize that ADA may play a role in testicular embryological development/descent, and/or it is possible that toxic purine metabolites could interfere with this process.

In our population, we also identified a high incidence of acquired undescended testis (16%), with 87% of cases requiring orchidopexy. In a healthy population, acquired undescended testes are reported to occur in 1–3% of cases [8]. Acquired undescended testes have a different pathogenesis compared with congenital undescended testes [7], mainly related to adhesions or increased stiffness/shortness of anatomical barriers involved. It is possible that metabolic abnormalities related to ADA deficiency could alter the histologic structure of these tissues. The toxic effect of ADA metabolites has been reported on different tissues, and it is well described how purinergic signaling plays an important role in fibrosis damage of several organs (skin, heart, liver, and lung) during tissue repair. For example, the profibrotic role of ADA deficiency in the lung has been clearly shown in an animal model with adenosine deaminase-deficient mice developing adenosine-dependent pulmonary fibrosis due to accumulation of ADA metabolites [10, 11]. We can hypothesize that ADA deficiency could cause fibrosis in tissues that are crossed by testes, increasing the stiffness of the physiologic anatomical barriers.

In our patients receiving PEG-ADA ERT, BMT, or GT (with or without conditioning), FSH was not elevated. Thus, in our sample, neither ADA deficiency nor the treatments received negatively affected pubertal development or gonadic function. We did not perform specific tests to evaluate fertility in our cohort, mainly due to the young age of the patients. We can assume that our patients have functional endocrine regulation of puberty as they have normal pubertal development and normal testosterone or estradiol levels. The oldest patient is 30 years, but the mean age of the group is 19 years. However, we cannot know whether a dysfunction of endocrine gonadal component will have a later onset. No data are available in the literature regarding fertility in ADA-SCID. For patients undergoing BMT, there is a risk of infertility which of infertility is higher (> 80%) in patients treated with conditioning regimens containing TBI, high-dose cyclophosphamide, melphalan, and busulfan. The use of a reduced-intensity conditioning regimen is expected to decrease HSCT-related side effects. Recently, the Pediatric Diseases Working Party of the European Society for Blood and Marrow Transplantation has established recommendations for the diagnosis and pre-emptive procedures that should be offered to all children and adolescents in Europe who undergo life-saving allogeneic SCT [12]. Emerging reports describe fertility and gonadal function in transplanted SCID [13–15], but actually, no specific studies on ADA-SCID have been performed. We recommend that these aspects deserve special attention considering the systemic manifestations of the condition (ADA-SCID) and the potential effects of its treatments on gonadal function.

In the literature, excess of adenosine in murine penile erectile tissues has been described associated with priapism [16]: This study highlights how adenosine deaminase plays a biological role in different tissues and systems. Considering our sample’s age, we did not analyze the erectile dysfunction.

The major limit of this report is the number of patients evaluated: We recognize that this study is based on limited sample size, but it is expected considering that ADA-SCID is an ultra-rare disease (from 1:200,000 to 1:1,000,000 births).

## Conclusion

In summary, this report describes the high incidence of urogenital abnormalities in a cohort of male ADA-SCID patients, which likely represents systemic manifestations of ADA-SCID. We identified a high incidence of cryptorchidism in our male patients with no urogenital abnormalities noted in females. Spontaneous and age appropriate pubertal development occurred in most females and males with a few cases of precocious or delayed puberty noted. We recommend regularly evaluating pubertal state as part of the complete physical examination in ADA-SCID patients. If cryptorchidism is present, we suggest undertaking specialist urologic evaluation as soon as possible. Patients with cryptorchidism have an increased risk of progressive infertility, testicular malignancy, and torsion [8]; successful relocation of the testes may reduce these potential long-term sequelae. Considering the impact urogenital and pubertal abnormalities can have on patients’ quality of life, we feel it is essential to include relevant history taking, clinical examination, and endocrine investigations in ADA-SCID patients to detect any abnormalities, initiate early treatment, and prevent long term complications.

## Electronic supplementary material

ESM 1 (DOCX 18.5 kb)

## Abbreviations

ADA: Adenosine deaminase
ADA-SCID: Severe combined immunodeficiency due to adenosine deaminase deficiency
PEG-ADA: Polyethylene glycol-conjugated adenosine deaminase
GT: Gene therapy
BMT: Bone marrow transplantation
HH: Hypogonadotropic hypogonadism
LH: Luteinizing hormone
FSH: Follicle-stimulating hormone
