# Supplementary material for: Urogenital Abnormalities in Adenosine Deaminase Deficiency
Source: J Clin Immunol. 2020 Apr 19;40(4):610–8. doi: 10.1007/s10875-020-00777-8 (PMC7253380; doi:10.1007/s10875-020-00777-8)
Supplement: Supplementary file 1 — (DOCX 18.5 kb) [file 10875_2020_777_MOESM1_ESM.docx]

Table 1S Female sample: urogenital abnormalities at pelvic US scan, pubertal development and hormonal tests

| N° | Pelvic us scan | Other urogenital disease | Pubertal stage | Precocious puberty | Treatment with GnRH agonist | Delayed puberty | Hypothalamus-pituitary-gonads axis |
| --- | --- | --- | --- | --- | --- | --- | --- |
| 1 | ND | No | Pubescent 18y TS V RM | No | No | no | ND |
| 3 | Normal | No | Pubescent 15y TS V RM | No | No | no | ND |
| 4 | Normal | No | Pubescent 12y TS V RM | Yes (8y) | Yes (8 - 11y) | no | ND |
| 8 | Normal | NA | NA* | Na | NA* | NA* | NA* |
| 13 | Normal | No | Pubescent 10y: B4 P2-3 | No | No | no | Physiologic activation |
| 17 | Normal | No | Prepubescent 3y | No | No | no | ND |
| 20 | Normal | No | Pubescent RM | No | No | no | Physiologic activation |
| 21 | Normal | No | Pubescent IM# | Yes (8y) | No | no | Physiologic activation |
| 26 | ND | No | Pubescent 13y: TS V | No | No | no | ND |
| 29 | ND | No | NA | No | No | No | ND |
| 30 | ND | No | Prepubescent 8y | No | No | No | ND |
| 32 | ND | No | Pubescent 17y | No | No | No | ND |
| 34 | ND | No | Pubescent IM | No | No | No | ND |
| 35 | ND | No | Pubescent at 15y, RM | No | No | No | ND |
| 37 | ND | No | Pubescent at 16y, RM | No | No | No | ND |
| 38 | ND | No | Pubescent at 15y | No | No | No | ND |
| 42 | Normal | No | Pubescent 14y, RM | No | No | No | Physiologic activation |
| 45 | Normal | Polycystic kidney disease | Pubescent at 15 y, RM | No | No | No | ND |
| 47 | ND | No | Prepubescent at 11y | No | No | No | ND |
| 51 | ND | No | Prepubescent at 10y | No | No | No | ND |
| 53 | Normal | No | Pubescent at 9y: A1P2B3 | Yes (9y) | Yes (9y-ongoing) | No | Normal |
| 54 | ND | No | Prepubescent at 10y | No | No | No | ND |
| 58 | ND | No | Prepubescent at 7y | No | No | No | ND |
| 60 | ND | No | Prepubescent at 4y | No | No | No | ND |
| 65 | ND | No | Prepubescent at 5y | No | No | No | ND |
| 66 | ND | No | Prepubescent at 5y | No | No | No | ND |
| 69 | ND | No | Prepubescent at 5y | No | No | No | ND |
| 72 | ND | No | Pubescent 13y, RM | No | No | No | ND |
| 73 | ND | No | Prepubescent at 2y | No | No | No | ND |
| 76 | ND | No | Prepubescent at 3y | No | No | No | ND |
| 79 | ND | No | Prepubescent at 2y | No | No | No | ND |
| 80 | ND | No | Prepubescent at 2y | No | No | No | ND |
| 83 | ND | No | Prepubescent at 4y | No | No | No | ND |
| 85 | ND | No | Prepubescent at 1y | No | No | No | ND |
| 86 | ND | No | Prepubescent at 1y8m | No | No | No | ND |

All female patients are reported in this table.

**Legend**: **NA**= not applicable. **ND** = not done. **UNK**= unknown. **TS** Tanner Stage. **RM** regular menses. **IM** irregular menses.

In the column “precocious puberty” the age of onset is reported in brackets

* patient n°8 is affected by Congenital Adrenal Hyperplasia with virilization of external genitalia and parents chose to maintained male external genitals. Not included in pubertal evaluation.

# patient n° 21 presented irregular menstrual cycles with prolonged periods of amenorrhea associated with hyperinsulinism, hirsutism and hyperandrogenism. Polycystic ovary syndrome was suspected, and the patient was treated with Cyproterone acetate and transdermal estradiol.
